# Scarification of the Gums in Dentition

**Published:** 1869-12

**Authors:** 


### EDITORIAL DEPARTMENT.

*Scarification of the Gums in Dentition.*—At a meeting of the "Edinburg Obstetrical Society" Dr. Cairns gave the following views on this subject, which we present in full to our readers, as they appear in the *Edinburg Med. Journal*:

I. Is scarification in dentition productive of any beneficial result? If it is so, in what do it good effects consist? The advantages alleged to accrue from the operation, as contained in the several works which I have consulted, may all be summed up in the following:—first, the relief of local pain; and, second, the prevention and arrestment of convulsions, laryngismus stridulus, diarrhœa, etc., etc.,

1. Scarification, according to its supporters, relieves local pain. Conceding meanwhile that this assertion is true, let us inquire into the grounds on which the assertion rests. Now it certainly cannot rest on the declaration of the little patients on whom the operation is performed because they have not yet acquired the power of speech—a circumstance indeed which renders the treatment of the diseases of children in general of a very difficult and unsatisfactory nature, preventing them as it does from correctly indicating either the precise seat of their sufferings, or the actual effects of the remedies employed. Well, if the allegation is not, and cannot be founded on the ground I have mentioned, it must in these circumstances be altogether and entirely of an inferential character. Now, the value of inferences is purely determined by the character of the data from which they are drawn. If the data are true, the inferences may be valid or they may not; but if the data are not true, the inferences must, as a matter of course, be utterly worthless. In the present case, then, what are the data from which it is inferred that scarification is productive of relief from pain? These data will, I think, be found on inquiry to consist in the tense, tumid, and congested condition of the gums. The matter stands thus: the gums, in the process of dentition, being in a tense, swolled, and inflamed state, are painful; and by relieving the tension, tumidity, and congestion by means of incisions, you thereby relieve pain. This, I opine, is a correct and fair statement of the case. Well, now, I demur entirely to the alleged fact. that in the *ordinary* process of dentition the gums are either tense or swollen. Is is quite true that there exists over the site of the approaching tooth an evident fullness: but this condition is caused, in all ordinary cases, by the presence of the tooth itself. The tissue overlying the tooth is not put into a state of strain by the tooth as the term tensity would lead one to suppose. No such thing; against such tension nature makes full and ample provision, by causing the subjacent gum to undergo gradual absorption in proportion to the growth of the tooth itself. The tooth is not *pushed* up. it *grows* up; and as *it* increases in growth, so do the overlying tissues become absorbed, thereby rendering tension impossible. Neither is there swelling in the ordinary sense of that term, because nature guards effectually against the infiltration of serum, by causing the growth of the tooth to be sufficiently slow, so as to give the vessels concerned abundant time to accommodate their calibre to the circumstances by which they are surrounded; and if a true swelling does in any case actually form, that is to be regarded simply as an accidental occurrence, and to be treated, of course, as it would be in ordinary circumstances, but it is in no wise essentially connected with the process under consideration. If, therefore, there is neither tension nor tumefaction, scarification is useless as a means of relieving pain, so far as regards the alleged disturbing influences of these two conditions. But what of inflammation? Simply this, that by abstracting blood from an inflammed part, you do not in the least degree either reduce or modify the inflammation. The part continues to be as red, as hot, and as painful as before. Nor do I hold it of much consequence to be told that the child has become more quiet after the operation, and must therefore have obtained relief by its means; because, unless its advocates are prepared to prove the result to be invariable—which they are not—I am fully entitled, in the circumstances, to assume, that such relief may have followed in spite of the operation, just as many patients have been found to recover from certain diseases in spite of the very questionable treatment to which they may have been subjected.

2. Scarification is alleged to prevent and arrest convulsions, etc.

Now, as a prophylactic remedy, the operation can only admissible under certain conditions :—1*st*, On the ascertained fact, that convulsions are an invariable accompaniment of dentition, 2*d*, That the operation uniformly, or at least generally, prevents their occurrence. The question, therefore, is, do these conditions hold ? I affirm they do not, and on the following grounds ; because convulsions, so far from always coexisting with the process of denti tion, do so in reality in a very small proportion of cases. They constitute, in fact, not the rule, but the exception. And further the object sought has in general not been attained ; that is to say, convulsions have just as frequently followed as they have preceded incisions of the gums. So much for the preventive ; and as regard the alleged curative agency of scarification, several questions naturally suggest themselves :—

(1.) Does it necessarily follow that dentition is the real exciting cause of the convulsions, merely because the latter happen to be concurrent with the former ? Every one, I daresay—even the most zealous advocate of the operation—would unhesitatingly answer in the negative, when the question is put in this pointed and direct manner ; nevertheless, I am rather inclined to think that there exists in the minds of most practitioners a strong predisposition to attribute every case of convulsions which occurs in a child within two years old to the so-called cutting of a tooth, and to that alone, unless other causes are so manifest as can hardly escape notice. Nor is the reason of this far to seek ; for, in the first place, it is universally admitted by every member of the profession, that dentition may, and does occasionally, induce convulsions ; in the second place, there exists a strong tendency in the human mind to connect certain effects with their most commonly received causes, whether true or false, and this circumstance has always operated in a very special manner in the minds of medical men.

(2.) A second question which suggests itself is, Has a recnrrence of the convulsive fits, which happen to take place during dentition, always been prevented by scarification ? An affirmative answer to this question would justly be held quite conclusive, at least as regards the particular circumstances referred to ; but unfortunately, I have not been able to find any one, within the compass of the research which I have made, who ventures to give the desiderated answer. On the contrary—unlike those who dogmatically proclaim, as an infallible remedy for this and that disease, this and that specific, which no other than themselves have ever been able to verify—even the most strenous supporters of scarification allege nothing more than simply that after the operation has been performed, the convulsions have ceased to recur only now and again.

(3.) And this brings us to a third question ,viz., .Whether, in those cases in which convulsions have ceased after the application of the lancet to the gums, the use of this instrument is to be regarded as the real procuring cause of their arrestment? Now, I do not by any means venture to say that it is not. This were too audacious by a great deal; but I do say, and without the least hesitation, that there exist more abundant data from which to give an answer in the negative than there do from which to give one in the affirmative. What, we ask, are the grounds on which the scarificator is employed? Because, say its advocates, after being applied, convulsions occasionally do not occur. And that is really the only answer which can be given. Very good; but when they are again asked, if they cau affirm with certainty that the use of the lancet has been the actual and sole means of stopping the convulsions, they feel obliged to be somewhat more cautious in the answer which they give. Their reply then is, It may be, or it may not be—we cannot absolutely say which. Well in these circumstances, we must be excused for expressing our humble opinion that the greater probability is, that it has not been so; first because the use of the lancet has just as frequently been followed by the *recurrence* of the convulsions as by their *discontinuance;* second, because their non-recurrence may have been a *mere matter of coincidence and nothing more.* It is well known for example, that in different children convulsions differ, both as regards their number and duration. In one child there is often only one convulsive attack, sometimes of short, and sometimes of considerably long duration; in another, we often find two, the one either following the other in close succession, or at a longer interval. Sometimes we find three, and so on; but when they are dependent on dentition, or other local irritation, they always prove of a self-limiting character. Suppose now, that in either of these cases you incise the gums, and that, after doing so, the convulsive attacks cease to return, are you entitled to give the credit to the lancet? If you say yes, I maintain that in the circumstances I am equally entitled to say no; because in all probability the convulsions had entirely ceased before the gums had even been touched by the lancet.

The same arguments which have been employed in the case of convulsions apply equally to the other diseases which I have mentioned as concurring with dentition, and, therefore, I may pass them over without further notice, merely adding that, although diarrhœa is perhaps one of the most common comitants of dentition, it seems somewhat strange that scarification should be so seldom practised, or even recommended for arresting that most debilitating of all the ailments to which infants are liable.

II. Having considered the beneficial, I now proceed to notice, in the second place, the prejudicial effects of scarification.

1. And here I allege, in the first place, that it is injurious, because it impedes the process of dentition. During the last few days, I have asked several professional brethern with whom I have come in contact, who approve of the operation in question, for what reason they do so? and the gist of the answer which I have received from each has been this: "Because," say they, "the lancet does at one stroke what nature would require a considerable time to accomplish to let the tooth through." And this quite accords with what we find in some of the books. Now, we aver the opposite. We aver that the use of the lancet, instead of rendering dentition more easy, makes it in reality more difficult. And here we must observe, that, in scarifying the gum, three different modes have been recommended—1*st*, by making a single incision; 2*d*, by making a crucial incision; and 3*d*, by making an elliptical incision, and removing that portion of the gum which overlies the tooth. Well, if either of the first two methods is adopted, in nine cases out of ten you have speedy reunion of the lips of the wound, thereby leaving matters exactly as they were before. If, as recommended by some, you go on repeating the incisions, you have just the same result following; thus rendering it extremely difficult for us, at least, to perceive how the approach of the tooth can be facilitated in the least degree by these means; while at the same time, the hard cicatrix which has been formed must require longer time to become absorbed as the tooth approaches than the soft natural tissue of the gum. If the wound heals by ulceration,—and by this process it must do so, when the third method is employed,—you do certainly obviate thereby the absorption of the gum, and thus seem to assist nature. But this, after all, is more apparent than real; because absortion is undergone not only in that portion of the gum which lies over the summit of the tooth, but also in the portions towards its sides,—portions, be it observed, which are left altogether untouched. But even although these portions were also removed, the truth of our averment would, in our opinion, be only strengthened thereby; and in this way, because you would thus expose a greater portion of the tooth to atmospheric influence,—premature exposure to which, by the removal of its natural covering, would give a material check to its growth and development. Consider, also, that by the operation, simple though it seem, you give a greater or less shock to the nervous system of the infant,—

and it is universally admitted that an infant at this period is in a state of high susceptibility, that you excite more or less inflammation, thereby increasing the suffering and irritability of the little patient; that you cause the loss of a certain quantity of blood, of which a child is highly intolerent, and particularly those children on whom the operation is performed, being generally of delicate and strumous habits; that you aggravate the painful condition of the gums, thereby rendering sucking a difficult operation, and preventing the infant from obtaining a proper supply of nourishment. Consider we say, these circumstances and the injurious effects which they must necessarily produce on the general constitution, and through it on the growth of the teeth, rendering that process, as they must do, unusually tedious and slow.

2. We allege, in the second place, that it may lead to fatal hæmorrhage. We are not in a position to state how often this result has followed from the operation; but if all the cases which have occurred had been recorded, and were collected, they might be found to amount to no inconsiderable number. At all events, it is well known that such cases have occurrd, and, indeed, it is only very recently that a case of this nature was reported to this Society by one of its members. To this, however it may be objected—1*st*, That in those cases in which the child has died from loss of blood, the incision may have been made too deep; our reply is that the incision is recommended to be made deep, so deep as to reach the tooth. 2*d*, It may be objected, that fatal cases may only have occurred in those children which happened to have the hæmorrhagic diathesis: we answer, that even although this were granted, you cannot discovcr whether this diathesis is present or not, until you make the incision, when the discovery is too late. 3*d*, It may also be objected, that the risk alluded to occurs so seldom that it need not act as a deterrent; to this we reply that the untoward results under consideration having happened even once or twice, renders it at least possible that it may also occur in the very case in which you are about to operate; and moreover should it do so and should you tell the parents on inquiry that you were aware that such an event might possibly occur, I rather fear that the parents would not hold you altogether blameless in the matter, and that they would bear you a secret grudge ever after.

3. I allege that it tends to perpetuate a custom which, to say the least of it, is of a doubtful character Probably one of the main reasons why the operation is so generally performed is, not in reality from the good effects which are expected to ensue from it, but because it is usually done in such circumstances. Others do it, and, in order not to appear singular or culpable, I must conform to the general practice, whether the issue should prove favorable or the reverse. In this way did the treatment by blistered, bleeding and violent drugging become transmitted from generation to generation,—age after age—, producing, as it is now universally allowed to have done, the most direful results. And in the same way has been handed down the operation in question, which though uncertain and doubtful in its results, continues to be in high favor and general use as a time-honoured custom. On this point, however, we do not enlarge, but proceed, as was proposed, to inquire.

III. If, in the circumstances, scarification is justifiable? We allege that it is not. 1, Because it inflicts unnecessary pain. The objection, observe, is not grounded on the fact that pain simply is caused to the child. Such an objection were absurd; because, although the medical practitioner holds it to be one of his prime functions to relieve pain, in many cases he can only fulfil that function by employing remedies which are themselves of a pain-giving nature. But this is not the question. The question is, am I warranted in employing a remedy which, so far as can be ascertained, does not relieve the pain which it is intended to do, and which remedy is itself painful both in its application and results? I maintain that in these circumstances I am not justified in doing so, and particularly when I remember the effects which scarification on one occasion produced in my own person. For it so happens, that when some years ago, my last wisdom-tooth was making its appearance, the late Professor Miller, at my own urgent request applied the lancet over it, but the result was, that instead of experiencing relief from the operation, it kept me, on the contrary, in a state of the most extreme suffering for days to come; the remedy, in short, having proved a thousand times worse than the disease.

2. It superinduces some of those very conditions which it professes to remedy. I allude in particular to tension, tumefaction, and inflammation, the relief of which, it will be remembered, was alleged as a reason why scarification should be performed. On that occasion, I simply endeavoured to show that the treatment recommended, had no rational grounds on which to rest; I now go a step further, and aver that scarification actually produces these results. Inflammation it must and cannot but excite; because, in virtue of a well-known physiological law, wherever you occasion a breach in living tissue. more or less inflammation results in order to repair the breach which has been made. Again in an inflamed part there is always more or less swelling, owing to the pressure upon the veins, which causes the exudation of serum into the surrounding cellular tissue. And, lastly, there is tension; because whether the scarified part heals by the first or second intention, there is in either case, contraction of the tissue, and consequent tension, if an unyielding structure like the tooth lies underneath.

I shall not be so bold as to affirm that scarification actually excites convulsions; but, considering the extreme sensitiveness of the gums, and the highly nervous condition of the child in some cases of teething, I do think that that operation is abundantly sufficient to act as an exciting cause of them. And it is certainly a fact, that there are some parents who will not allow the gums of their infants to be incised on any account, because in the case of former children, they have observed the operation to be followed by convulsions; and parent are very acute and often very correct observers in refference to the ailments of their children—a fact which renders their testimony in such matters of no inconsiderable value.

3. At the best, it is a mere experiment. This, I think, cannot be denied, with whatever view the operation may be performed, whether to relieve pain, or whether to arrest convulsions, or any of the other symptoms which have been mentioned as coincident with dentition. If you perform the operation to relieve pain, you do so simply as an experiment, because, in the first place, you do not know if the pain from which the child appears to suffer is due to the state of the gums at all; it may depend upon causes totally different. In the second place, granting that the gums *are* the prime source of the irritation, how do you know that the *part* of the gum which you incise is the real seat of the pain? You perceive a certain portion of the gum to be somewhat prominent, and find at the same time that the child gives certain expresssions of suffering and you thereupon immediately leap to the conclusion that the pain is occasioned by that particular part of the gam. Are you *certain* that it is so? You are not; you cannot be. The *greater* probability is, that the irritation is entirely due to the growth of *a* tooth, which, owing to the early period of its development, gives no indication whatever of its appearance. In the third place, even although you could hit exactly upon the precise tooth which caused the pain, how do you know whether it is the *superficial* or radical part of the tooth which gives rise to the pain? Whoever has suffered from tooth-ache, must know that the pain in *many* cases arises from the *root* of the tooth, and not from the crown, showing that the former is just as likely to be the seat of pain as the latter; and consequently, that in scarification, the object sought will most probably prove altogether abortive. and therefore out-and-out experimental.

And as regards convulsions, etc., scarification of the gums is a thousand times more doubtful in its results than as regards the relief of pain. Who can deny on how many occult causes such phenomena may actually depend? But simply because a child happens "to be getting its teeth" while a convulsive fit occurs, the convulsion is at once attributed to the state of the gums. The gums are forthwith lanced, and if the convulsions cease, the lancet gets the credit; if they do not cease, *as in general they do*

*not*, the lancet nevertheless is extolled as having done all that could have been done to avert bad consequences. But now, al, lowing scarification to be nothing more than an experiment, is it or is it not justifiable ? To this I reply, that it is only justifiable on certain grounds. An experiment is not justifiable, 1*st*, When there is no essential connexion between the disease and the alleged cause for the removal of which the experiment is made. 2*d*. When it has repeatedly failed to produce the desired result. 3*d*. When it is likely to be more injurious than beneficial. These points, however, I simply state without enlarging upon them, having greatly exceeded the limits to which I had restricted myself."

While we are not prepared to endorse all of the views of Dr. Cairns, which we have laid before our readers in full, yet at the same time we are no advocates of indiscriminate scarification of the gums of children during first dentition. We can hardly suppose it possible for any one who has had experience in this matter to deny that the lancing of tumefied gums is a relief ; or that convulsions may be prevented by this operation.

While it is true that it is a question with many whether the too early scarification of the gums does not impede the eruption of the teeth, from the cicatrix resulting offering more resistance to the advancing tooth than the gum in its original condition, at the same time we had supposed that every one would admit the relief it affords to the tension exerted in the tumefied part.

If we deny that the irritation of dentition is capable of producing convulsions, then Dr. Cairns' theory that scarification does not arrest these nervous disorders but aggravates them, may be a correct one.

It is true, fatal results have attended some cases where this operation of lancing the gums has been performed, but in such cases only where the child has afforded unmistakable evidence of a hæmorrhagic diathesis, as is shown by blood deficient in fibrine and red globules, and consequently wanting in the power of coagulating ; or a condition of blood due to the action of mercury —in other words impoverished.

For we know that mercury will decompose blood ; and Dr. Wright, who has analysed the blood of patients under mercurial action, states that it is materially changed, containing more water, and being more prone to putrefaction than healthy blood, the destructive agency depriving it of one-third of its fibrine, one-sixth or more of its globules, and at the same time loads it with a fœtid matter, the product of decomposition.

In our opinion Dr. Cairns has advanced nothing which proves the operation of scarification a dangerous and useless one in all cases ; but on the contrary we consider it one of great benefit in the vast majority of cases where the proper indications exist for its performance.

